# Medical Speculations

**Published:** 1804-05-01

**Authors:** 


### *To the Editors of the Medical and Physical Journal.*

GENTLEMEN,

SINCE I first applied myself to the study of Medicine, I have been accustomed to note, in a kind of Common-Place-Book, such ideas and observations as I conceived to be new and peculiar to myself, and likely to prove interesting in their consequences; consisting chiefly of projects and speculations, tending to the improvement of practical Medicine. I review them periodically, and expunge such as further reading or conversation teach me to be unimportant, or to have occurred previously to another. A few remain, which, if you think them worthy of public notice, I offer for insertion in your very valuable Journal. I could name not a few instances, in which; from a reference to dates, I see clearly that I might have claimed claimed to myself the full and uncontested merit of having first thought on projects, which have proved to others a source of very favourable reputation; but my speculations were confined to my Note-Book, and I have no reason to complain. Some of my projects may, possibly, have already occurred to others, may have been published perhaps; I shall not, be assured, in any one case, knowingly arrogate to myself the property of another. My communications, indeed, (for I mean, with your permission, to occupy a page or two of your interesting publication occasionally) will not, I fear, be such as medical men would choose to acknowledge, or of which they would think it worth while to dispute the possession.—Once for all, however, I commit to your hands the full power of withholding such part of them as you may deem either not new, or not sufficiently interesting for publication.

Should it accord with the Plan you have proposed to yourselves for the conduct of the Medical and Physical Journal, to admit anonymous communications of this nature, I am not without hopes that others, possessed of more talent and experience than myself, may be induced, from time to time, to add to the Stock of

### Medical Lucubrations and Speculations.

1. *Submersion in Hydrophobia.* Many years have elapsed since it was proposed to immerse a person, labouring under hydrophobia, in water, so as almost to drown him. I would go further, I would actually keep him under water until he should have ceased to move, until the entire of the functions seemed to be suspended; but I would have at hand all the modern resuscitative apparatus of the best kind, Galvanic troughs, an electrical machine, &c. to be employed as soon as the superintending physicians should think proper. Death might ensue, it is granted; but if I had the misfortune to suffer, in my own person, from *rabies canina,* I should, most certainly, wish to have this chance (such as it is) of recovery. Does any known mode of treatment present a better? We meet with some dreadful and desperate cases of tetanus and melancholia on record, in which it might have been excusable to try such an experiment.

2. *Inoculation of the Measles.* I am not altogether ignorant of what has been, heretofore, published on this subject; but I think that I see a new and a more certain source of morbillous virus. I would rub in, assiduously, on the breast, on the day just preceding that on which

 the the eruptions would be expected to appear, a little of the unguent. antimon. tartarisat. in such manner that the pustules, excited hereby, might come forward and be blended with the exanthematous efflorescence of the measles I would inoculate with the fluid contained in these pustules. No harm could, I believe, ensue; and the party from whom the virus is procured, would, probably, be benefited by the irritation of the surface of the breast.*

3. *On the Extinction of the Small-Pox.* I have doubts how far it is advisable to attempt its utter extinction in this country, unless our continental neighbours, or, I should say, unless the entire of the inhabitants of the globe co-operate with us; since it is *possible*, however earnest and general may be our exertions to exterminate it, that it may shew itself again in this country in the course of forty, fifty, or sixty years hence; since it is established that the susceptibility of any contagious disease is in an inverse ratio to the frequency of exposure to its contagion; and since it is obvious, from the bills of mortality and the history of the small-pox, that the air of Great Britain has not been, at any one time from the first introduction of the small-pox among us, entirely free from its effluvia, to which we are all, unknowingly, every day exposed: I argue that, on the re-appearance of the small-pox in Great Britain, with the interval above supposed, the entire of the present generation may have acquired a new susceptibility; that these, who have had the Cow-pox, nay I would say, that those, who have actually had the small-pox, may not be exempt from its further ravages, under the above-mentioned circumstances. Have those persons, supposed to have had the small-pox *twice*, passed the intervals in places entirely, or in part, secluded from its effluvia, as *in the country*, or *at sea*? Need I adduce the case of the celebrated Genevese physician, Bonnet, in illustration of my argument? He had enjoyed considerable practice for nearly forty years, when he retired into the country. He had here passed about ten years, seldom, if ever, visiting the sick, when he was called to see a young man, ill of small-pox; he now took that disease, to which he was before exposed times innumerable with impunity. See Bibliot. German, vol. 3, Note, about the middle of the volume.

*Bristol, April* 10, 1804.

[ To be continued. ]

---

* This speculation appears to deserve attention; but if Dr. Blackburne's Theory (No. 57, p. 461) be true, the measles so produced will be attended with pustulary eruptions. Ed.

